# Two new risk factors for heterotopic ossification development after severe burns

**DOI:** 10.1371/journal.pone.0182303

**Published:** 2017-08-04

**Authors:** Laurent Thefenne, Gratiane de Brier, Thomas Leclerc, Claire Jourdan, Claire Nicolas, Stéphanie Truffaut, Eric Lapeyre, Francois Genet

**Affiliations:** 1 Service de Médecine Physique et de Réadaptation, Service de Santé des Armées, Hôpital d’Instruction des Armées Laveran, Marseille, France; 2 Centre de Traitement des Brûlés, Service de Santé des Armées, Hôpital d’Instruction des Armées Percy, Clamart, France; 3 Département de Médecine Physique et de Réadaptation-Thérapeutique, Centre Hospitalier Lapeyronie, Montpellier, France; 4 Service de Médecine Physique et de Réadaptation, Centre de Réadaptation de Coubert, Coubert, France; 5 Service de Médecine Physique et de Réadaptation, Service de Santé des Armées, Hôpital d’Instruction des Armées Percy, Clamart, France; 6 Service de Médecine Physique et de Réadaptation, Hôpital Raymond Poincaré, Garches, France; 7 Université Versailles Saint Quentin en Yvelines, « End:icap » U1179 INSERM, UFR des Sciences de la Santé – Simone Veil, Montigny le Bretonneux, France; National Yang-Ming University, TAIWAN

## Abstract

**Objectives:**

Life after severe burns is conditioned by the remaining sequelae. The pathophysiology and risk factors of Heterotopic Ossification (HO) after burns are still poorly understood. The aim of this study was to determine: 1) the incidence of HO after burns and 2) the risk factors associated with HO development, in a large retrospective study.

**Methods:**

A case-control study of patients admitted to the burns intensive care unit of Percy Hospital, Paris, from the 1^st^ January 2009 to the 31^st^ December 2013 and then admitted to one of three centres specialised in the rehabilitation of patients with burns. Multivariate analysis was carried out to analyse the relationship between HO development and demographic and clinical data.

**Results:**

805 patients were included. 32 patients (4.0%) developed a total of 74 heterotopic ossifications, that is a little higher incidence than the incidence found in the literature. The epidemiological characteristics of the population studied was similar to the literature. HOs were mainly localized around the elbows, followed by the hips, shoulders and knees. Each case-patient was paired with 3 control-patients. There were significant associations between HO development and the length of stay in the burns intensive care unit, the extent and depth of the burns, the occurrence of pulmonary or cutaneous infections, use of curare and use of an air-fluidized bed.

**Conclusion:**

In addition to recognized risk factors (duration of stay in the intensive care burns unit, extent and depth of burns, pulmonary and cutaneous infections), the use of curare and the use of a fluidized bed (with the duration of use) were significantly associated with HO formation.

## Introduction

**Fig 1:**
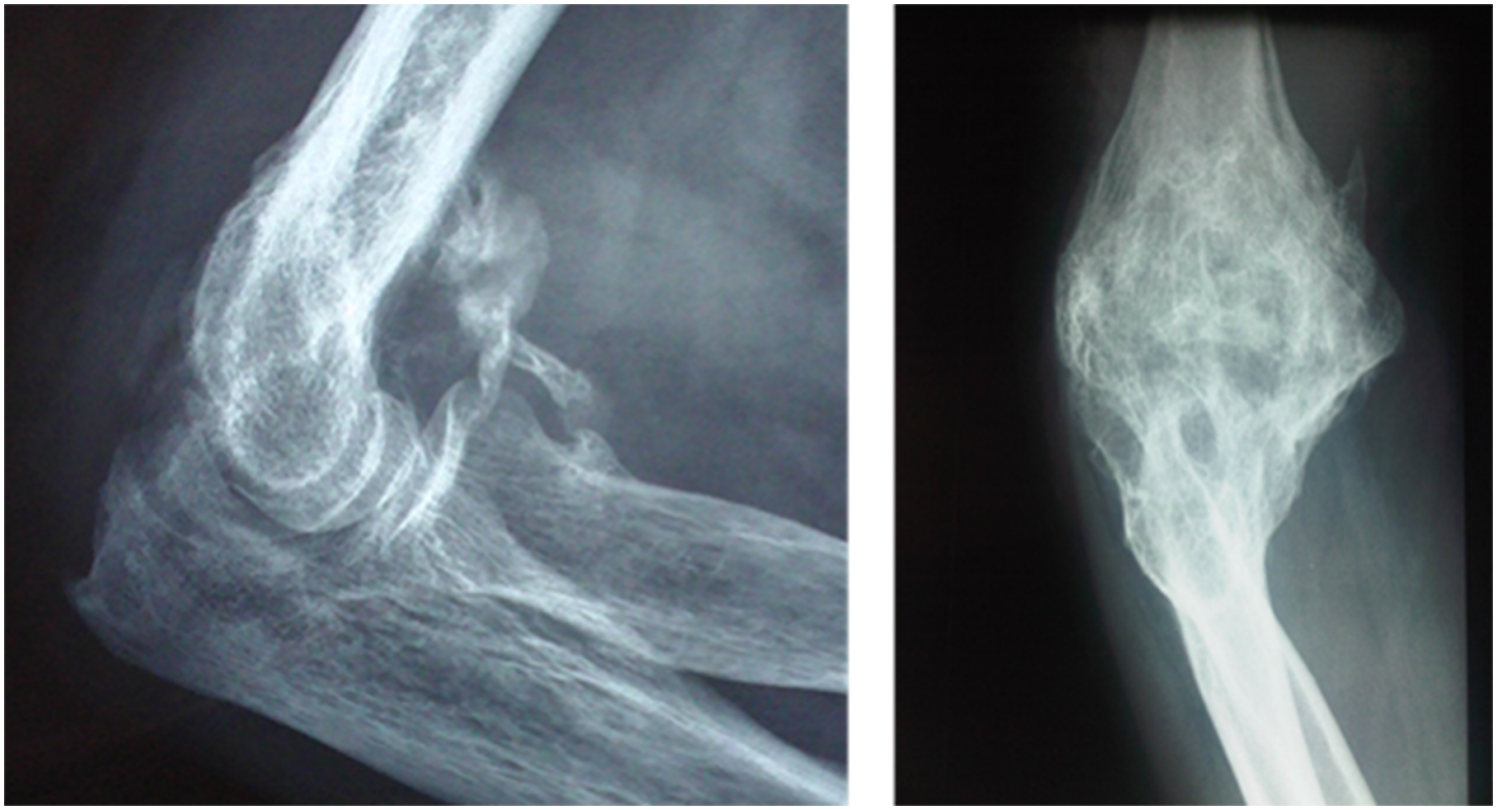
Heterotopic ossification around the elbow in patients with burns. A and B: X-ray images of circumferential HO.

After severe burns, the future and quality of life of patients is largely dependent on neuro-orthopaedic sequelae. One complication is the development of heterotopic Ossification (HO), the benign formation of peri-articular lamellar bone [1]. As well as in the case of burns, HO can occur in a variety of conditions including following orthopaedic trauma, central nervous system lesions and genetic diseases (fibrodysplasia ossificans progressiva) [1]. HO reduces the range of motion of the affected joint and may limit function, depending on the joint affected (Fig 1), and can compress nerves and vessels [2].

HO was first described after spinal cord injury by Augusta Klumpke Dejerine and André Cellier in 1918 [3]. The first description of burns-related HO was in 1930 by Lloyd [4,5]. HO generally develops several weeks after the burns, while patients are still in intensive care. The prevalence of HO is relatively low (about 1–3%) [6], however it can cause major functional limitations in affected patients. Several factors relating to the stay in intensive care, such as infections, prolonged inflammation and immobilisation are believed to influence the development of HO, along with the extent of the burns, however few studies have specifically investigated this question [4,6–9]. Moreover, the mechanism of HO development is not completely understood. Two hypotheses have been proposed. HO can occur in areas which are not burned, and since burns are rarely deep enough to reach joints [4], some authors have suggested that HO is induced by metabolic dysregulation, mediated by inflammatory and immune responses [4,10–12]. The second hypothesis is that mobilisation following immobilisation, or strong mobilisation may cause repetitive micro trauma, which is known to trigger HO formation [4,7,13]. In parallel, several studies have also described HO in patients who do not respond to physiotherapy [14,15].

The risk factors reported in the literature include the extent and depth of the burns (HO is mainly associated with deep burns), infections (particularly cutaneous), and strong mobilisation or periods of immobilisation [7,9,13,16].

The aim of this retrospective study was to determine: 1) the incidence of HO after burns in patients initially admitted to a recognized Burns Intensive Care Unit and 2) if previously reported risk factors are actually associated with the development of HO, in a large sample of patients.

## Material and methods

### Ethics statement

This was a retrospective non-interventional study in which usual procedures were carried out without any additional procedures (diagnosis or medical supervision). In France, patient consent is not required for such an anonymous retrospective data analysis. We confirm that this study was reviewed and approved by our Institutional review board (IRB) before the study began. Our IRB specifically stated that this study was conform to the regulations in course and did not need consent for this study [Comité de Protection des Personnes Ile de France XI, Saint Germain en Laye, France].

### Study design

The medical records of patients over the age of 15 years who were admitted to the burns intensive care unit of Percy Military Hospital with acute burns between January 2009 the 1^st^ and December 2013 the 31^th^ (805 patients) were analysed. Following the acute phase, patients who required rehabilitation were then admitted to one of three physical medicine and rehabilitation departments: CRF Coubert, La Musse and the Physical Medicine and Rehabilitation Department of Percy Military Hospital. The follow-up data (at least 3 months) were obtained via the Medical Databases of these centres.

A case control study design was used to compare the clinical features and medical history of patients with burns who developed HO with those who did not. HO was suspected when local inflammation and pain occurred around a joint and/or when joint range of motion became limited. A CT scan was then carried out to confirm the diagnosis. Each patient who developed HO was matched with 3 control patients (who did not develop HO). The control patients were randomly selected by the Medical Information Department manager. The matching criteria were: age, gender and type of burn (thermal, electrical, chemical or radiological). The following data were recorded for each patient: length of stay in the burns intensive care unit (days), the extent (%) and depth of the burn (1 to 3), any infections which occurred (cutaneous, pulmonary or urinary), use of curare (yes or no) with duration of use (days), and use of an air-fluidized bed (yes or no) with duration of use (days).

### Statistical analysis

Statistical analysis was carried out using “R” software version 2.10.1 (the R foundation). Descriptive data were calculated, including means, medians, minima, maxima, standard deviations, and percentages. A Chi^2^ test was used to compare normally distributed qualitative variables, a Wilcoxon test was used to compare normally distributed quantitative variables and a Fisher’s exact test was used for data that were not normally distributed. ANOVA was used to analyse continuous variables. All p values were 2-tailed and p<0.5 was considered statistically significant. A multivariate logistic model was then used, with the variables used for the stratification (age, gender and type of burn) as independent variables. Odds ratios and adjusted odds ratios were both calculated with a 95% CI.

## Results

**Fig 2:**
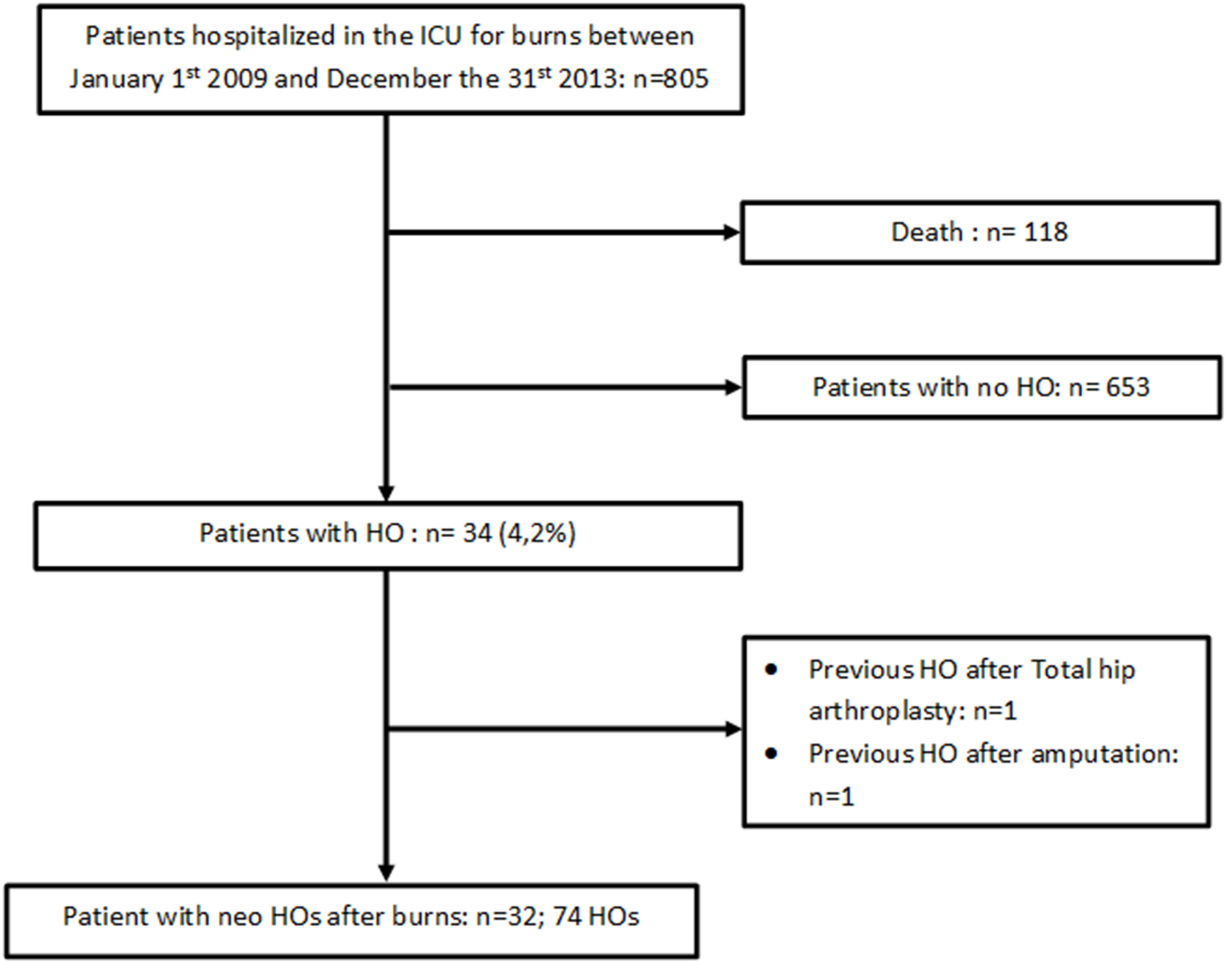
Flow Chart of patients’ files (all patients hospitalized in the ICU for burns between January 1^st^ 2009 and December the 31th 2013).

A total of 74 HOs developed in 32 of the 805 patients (Fig 2). Thus 4.0% of patients developed HO. The mean number of HOs per patient was 2.3 (range 1 to 7).

### Case series (Table 1)

**Table 1:** Characteristics of case and control patients.

		Cases: 32	Controls: 96
Gender ratio		20 men (62.5%)	60 men (62.5%)
Age	Mean	45.2 +/- 14.9 [16–75]	45.1 +/- 14.9 [16–77]
	Median	47 [35–56]	45.5 [33.75–58]
Type of burn	Thermal	30 (93.8%)	90 (93.8%)
	Electrical	2 (6.2%)	6 (6.2%)
	Chemical	0	0
	Radiological	0	0

Of the 32 patients who developed HO, 20 were men, gender ratio: 62.5%. Mean age at the time of the accident (burn) was 45.2 ± 14.9 (range 16.0 to 75.0). Thirty burns were thermal (93.8%) and 2 were electrical (6.2%). There were no chemical or radiological burns.

The primary location of HO was the elbow (37; 50.0%), followed by the shoulder (15; 20.3%), the hip (13; 17.6%) and the knee (8; 10.8%), with one case of HO at the wrist (1.3%). HO was suspected when there were limitations in range of motion, pain or local inflammation; it was confirmed by radiography. Patients admitted to La Musse (n = 73) underwent systematic x-ray of most joints and thus some non-symptomatic HO were detected. Of the 73 patients followed in La Musse, 13 (17.8%) developed 36 HOs. Twenty-two HOs (22/36; 61.1%; 4 patients) were symptomatic whereas 14 were not (14/36; 38.8%; 9 patients).

All patients were mobilised daily by a physiotherapist during their stay in the intensive care burns unit. No patients received non-steroidal anti-inflammatory drugs or bisphosphonates.

### Control series (Table 1)

Ninety-six patients did not develop HO (60 men, gender ratio: 62.5%), mean age at the time of accident (burn) was 45.1 +/- 14.9 (range 16.0 to 77.0). Ninety burns were thermal (93.8%) and 6 were electrical (6.2%). No patients had chemical or radiological burns.

### Univariate analysis (Table 2)

**Table 2:** Relationship between HO development and factors analysed.

	Cases	Controls	p
Length of stay ICU (days)	94.9	32.9	p<0.001
Mean total burn area (%)	48.5	18.2	p<0.001
Mean of deep burns (%)	33.1	7.9	p<0.001
Pulmonary infection (n)	27	28	p<0.001
Cutaneous Infection (n)	22	24	p<0.001
Urinary infection (n)	6	9	p = 0.24
Curare utilization (n)	25	14	p<0.001
Duration of use of curare (days)	4.9	1	p<0.001
Duration of curare use for patients that received it in both groups (days)	6.4	7.1	p = 0.8
Use of fluidized bed (n)	28	20	p<0.001
Duration of use of fluidized bed (days)	59	4.7	p<0.001
Length of fluidized bed use for patients that used one in both groups (days)	67.4	3.6	p<0.001

There was a significant relationship between length of stay in the intensive care burns unit, extent and depth of burns, occurrence of pulmonary and cutaneous infections and the development of HO. There was no relationship between urinary infection and HO. There was a significant relationship between use of curare, duration of curare use, use of a fluidized bed and duration of use, and development of HO. When comparing only patients that received curare (not all patients received curare in either group), the effect of the duration of the curare use was no longer significant.

### Multivariate analysis (Table 3)

**Table 3:** Main results of the multivariate analysis.

Factors analyzed	Cases	Controls	Adjusted Odds ratio	95% confidence interval
Length of stay (days)	94.9	32.9	1.1	[1.1–1.2]
Mean total burn area (%)	48.5	18.2	1.1	[1.1–1.2]
Mean of deep burns (%)	33.1	7.9	1.1	[1.1–1.2]
Pulmonary infection (n)	27	28	21.5	[6.0–77.4]
Cutaneous infection (n)	22	24	7.5	[3.0–18.6]
Urinary infection (n)	6	9	2.5	[0.8–8.1]
Curare use (n)	25	14	24.1	[8.3–70.5]
Duration of use of curare (days)	4.9	1	0.99	[0.9–1.1]
Use of fluidized bed (n)	28	20	39.6	[10.4–150.5]
Duration of use of fluidized bed (days)	59	4.7	1.1	[1.0–1.2]

After adjusting for the matching criteria, the relationship between the development of HO and duration of stay in the intensive care burns unit, extent and depth of burns, pulmonary and cutaneous infections, use of curare, use of an air-fluidized bed and the duration of use remained significant. The relationship with the duration of curare use was no longer significant.

## Discussion

### Main results

The incidence of HO after severe burns found in this study was about 4.0% (32/805). All the risk factors reported in the literature were statistically associated with the development of HO. However, the use of curare and fluidized beds (including the duration of use) were also associated with HO formation.

### Epidemiology

The last epidemiological study of patients with burns was carried out by the French Health Watch Institute (INVS, 2011) [17]. Their data were comparable with the data in the present study, except for mortality rate and duration of intensive care burns unit stay, which were longer in the present study. This is probably due to the type of patients included in each study. In the present study only patients who were admitted to the intensive care burns unit were included, whereas the INVS study included patients with burns admitted to any department (probably less severe lesions).

Of the 805 patients admitted with severe burns during the study period, 32 developed HO (4.0%). Since most patients had HO in more than one location (mean 2.3 locations, ranging from 1 to 7), there was a total of 74 HOs. Reports in the literature describe an incidence of HO of 1–3% in patients with burns [5,8,18]. This higher incidence may be related to the fact that some of the patients admitted to La Musse rehabilitation center underwent systematic radiographic screening. Nine patients with no symptoms were diagnosed with HO in this manner (13.7%, 36 HOs). There are no reports of systematic screening for HO in the literature. However, the patients in the present study had severe burns, which are known to be associated with a higher frequency of HO [4,7,9,16,18,19]. Only 24% of these HOs were symptomatic, suggesting the frequency of HO after burns is underestimated.

Concerning the location, 36 HOs (48.6%) occurred around the elbow. This is lower than other reports, which state that 80% of burns-related HOs are around the elbow [5,7,9,16,18]. This difference may be attributed to the fact that some of the patients in the present study underwent systematic radiographic screening, which changed the distribution of the locations of the HOs (by the inclusion of non-troublesome ones). In patients with central neurological lesions, the primary location of troublesome HO is the hip [1,20] followed by the elbow. We postulate that the higher incidence of elbow HO may be related to the higher incidence of elbow than hip burns, since local inflammation (lesion) is a major provocative factor for HO [21].

### Risk factors

The results showed a significant relationship between the length of stay in the intensive care burns unit, the extent of the burns, cutaneous and pulmonary infections and HO. These factors are frequently mentioned in studies of HO but had not been specifically studied [4,7–9,14–16,18,22]. One study found a significant relationship between wound healing time and the development of HO [23]. We did not analyse this factor because it was too difficult to determine, especially in a retrospective study. Orchard and al, in a recent retrospective case control study, investigated the risk factors for HO and found similar results to the present study: a significant association of percentage Total Body Surface Area and sepsis were with HO development [24]. Recently, in a large database of six high-volume burn centres, specific but cofounding (with the severity of the lesions) risk factors were identified: patients with burns covering more than 30% of total body surface, arm burns requiring skin grafting, each additional time a patient went to the operating room, and each additional day of ventilation [6].

### Immobilization and iatrogenic paralysis

Prolonged immobilization is suspected to be a significant factor for HO development [25]. Studies of the formation of HOs after central neurological lesions have shown that delayed access to a rehabilitation centre is a risk factor [25]. Moreover, the paralysis is also considered as a cofounding factor relating to the importance of early mobilization in ICU [25]. For example, this explains why HOs can occur in the case of peripheral nerve lesions such as Guillain-Barré syndrome [26]. It is for this reason that we chose to evaluate additional factors relating to immobilization that could have some effects on osteogenesis: the use of curare and fluidized beds.

Curare induces complete paralysis and muscle atonia, and the paralysis induced may be similar to that during the acute phase of cerebral or spinal lesions. This drug is notably utilized in ICU to control ventilation and help with intubation. Several previous studies have reported HOs following the extended use of curare [27–29]) and the present study also showed an association between curare and the risk of HO. This suggests that iatrogenic conditions could participate in HO development.

A fluidized bed induces muscle relaxation and global hypotonia, similarly to the effect of curare. Furthermore, the use of a fluidized bed has many secondary effects such as dehydration, inflammation and fever. It may also produce an effect of weightlessness, similar to astronauts, for example. Moreover, patients who are put on these kinds of beds have more severe conditions, which may be another cofounding factor. However, there are no studies of weightlessness and HO; the only known effect of weightlessness on bone is osteoporosis.

### Preventive treatments and mobilizations

Few studies have evaluated the prevention of HO following burns. One study evaluated the use of bisphosphonates, which inhibit calcification. The patients received Etidronate, 300mg, twice a day, from the second week of care to the end of care, however no effect was found on HO development [30]. Non-steroidal anti-inflammatory drugs (NSAIDs) could be useful to decrease the inflammation which is often associated with burns. These drugs have been shown to be effective in preventing HO in orthopaedic pathologies [31] and after paraplegia [32]. However, NSAIDs are often contraindicated in the case of infection, renal disorders and risk of bleeding. There is no data in the literature regarding the use of NSAIDs for the prevention of HO in rehabilitation centres.

The hypothesis that immobilization may be a risk factor for HO after burns, is further supported by the fact there is a significant relationship between HO development and the time before beginning active movement [24]. In parallel, following their experience, some teams suggest that physiotherapy may be the only uncontested method of prevention for HO [5,16]. Physiotherapy is also important for the prevention of skin contractures; however, it may be postponed because of surgery or cutaneous fragility. Relief incisions seem to increase the prevalence of HO, however, they are performed in patients with severe burns, and thus there are important confounding factors. The data from this study do not allow us to confirm that early passive mobilizations are a mean of preventing HOs for patients who are immobilized under curare and/or on fluidized beds after severe burns. Equally, the effect of the type of curare should be studied.

### Limitations

This was a retrospective study, therefore the collection of data was more difficult and some patients were lost to follow-up, limiting the long term analysis. Some of the factors analysed were related to each other, such as the extent of the burns, the length of stay and infections. It is thus more difficult to conclude regarding the influence of each individual factor on HO development. The inclusion of non-symptomatic HOs may have been inappropriate since no treatment was proposed. However, the main aim of the study was to determinate the epidemiology and risk factors for the formation of heterotopic ossifications. This is why non-symptomatic HOs were also included. Physiotherapy was not sufficiently described in the medical files, thus no data were available regarding mobilization procedures. Considering that forced passive mobilization may be an associated factor in HO development in severely burned non-communicating patients in intensive care units, the results of this study do not confirm that early mobilization is safe and can reduce the incidence of HO.

## Conclusion

The prevalence of heterotopic ossification after severe burns was about 4.0% in this study. In addition to recognized risk factors (duration of stay in the intensive care burns unit, extent and depth of burns, pulmonary and cutaneous infections), the use of curare and the use of a fluidized bed (with the duration of use) were significantly associated with HO formation. These results suggest that immobilization of patients with burns in ICU could be an associated risk factor for HO development. Conversely this study did not prove that early mobilization prevents ectopic bone formations mainly because of the lack of assessment of the intensity of the mobilization (retrospective design). Further investigations are necessary.

## Supporting information

S1 Table Database of the series of patients. (PDF)

S2 Table Database of the heterotopic ossification features. (PDF)
