# Supplementary material for: Two new risk factors for heterotopic ossification development after severe burns
Source: PLoS One. 2017 Aug 4;12(8):e0182303. doi: 10.1371/journal.pone.0182303 (PMC5544177; doi:10.1371/journal.pone.0182303)
Supplement: S1 Table — (PDF) [file pone.0182303.s001.pdf]

Case / Control	sex	type OF BURNS	HO (n)	Adressed by	Length of stay	TSSB %	TDSB %	Treated Pulm Infect	Treated Urin Infect	Treated Cut Infect
**1**	**1**	**1**	**7**	**1**	**194,00**	**70**	**50**	**1**	**1**	**1**
2	1	1	0	1	10,00	16	0	1	0	0
3	1	1	0	1	1,00	2	0,1	0	0	0
4	1	1	0	1	9,00	10	0	0	0	0
**5**	**2**	**1**	**1**	**1**	**57,00**	**56**	**30**	**1**	**0**	**0**
6	2	1	0	1	30,00	20	5	0	0	0
7	2	1	0	3	9,00	10	0	0	0	0
8	2	1	0	1	0,00	5	0	0	0	0
**9**	**2**	**1**	**1**	**1**	**114,00**	**52,1**	**34**	**1**	**0**	**1**
10	2	1	0	1	3,00	1,7	0	0	0	0
11	2	1	0	1	98,00	20	14	1	0	0
12	2	1	0	1	93,00	54,2	54	1	0	1
**13**	**1**	**1**	**1**	**1**	**47,00**	**20**	**20**	**0**	**0**	**0**
14	1	1	0	1	51,00	26	15	1	0	1
15	1	1	0	1	23,00	10	9	0	0	0
16	1	1	0	1	34,00	10	10	1	0	1
**17**	**2**	**2**	**2**	**1**	**69,00**	**55**	**40**	**0**	**0**	**0**
18	2	2	0	1	19,00	40	5	0	0	1
19	2	2	0	1	23,00	25	5	0	0	0
20	2	2	0	1	1,00	1	0	0	0	0
**21**	**2**	**1**	**1**	**1**	**118,00**	**65**	**55**	**1**	**1**	**1**
22	2	1	0	1	2,00	6	0	0	0	0
23	2	1	0	3	42,00	51	15	0	0	0
24	2	1	0	1	46,00	40	6	0	0	1
**25**	**2**	**1**	**5**	**1**	**111,00**	**40**	**25**	**1**	**0**	**1**
26	2	1	0	1	19,00	22	5	0	0	1
27	2	1	0	1	38,00	25	5	1	0	1
28	2	1	0	1	16,00	10	0	0	0	0
**29**	**1**	**1**	**4**	**1**	**96,00**	**33**	**23**	**1**	**0**	**1**
30	1	1	0	1	67,00	62	45	1	1	0
31	1	1	0	1	26,00	15	5	0	0	0
32	1	1	0	3	1,00	6	0	0	0	0

**33**	**1**	**1**	**3**	**1**	**168,00**	**50**	**40**	**1**	**0**	**1**
34	1	1	0	1	64,00	30	9,2	0	0	1
35	1	1	0	3	3,00	6	0	0	0	0
36	1	1	0	1	22,00	10	4	0	0	0
**37**	**2**	**1**	**1**	**4**	**3,00**	**25**	**0**	**MD**	**MD**	**MD**
38	2	1	0	4	37,00	27	8	1	0	1
39	2	1	0	1	28,00	20	10	0	0	0
40	2	1	0	1	32,00	25	7	0	0	1
**41**	**2**	**1**	**1**	**1**	**120,00**	**29**	**20**	**1**	**0**	**1**
42	2	1	0	1	98,00	30	15	1	0	1
43	2	1	0	1	34,00	25	5	0	0	0
44	2	1	0	2	39,00	18	6	0	0	0
**45**	**1**	**1**	**2**	**1**	**242,00**	**75**	**60**	**1**	**0**	**1**
46	1	1	0	1	1,00	4,5	0	0	0	0
47	1	1	0	1	41,00	30	30	0	1	0
48	1	1	0	1	33,00	15	6	0	1	0
**49**	**2**	**1**	**2**	**1**	**106,00**	**60**	**31**	**1**	**0**	**1**
50	2	1	0	4	13,00	8	0	0	0	0
51	2	1	0	3	21,00	8	0	0	0	0
52	2	1	0	1	31,00	10	2	0	0	0
**53**	**2**	**1**	**2**	**1**	**89,00**	**40**	**35**	**1**	**0**	**0**
54	2	1	0	1	59,00	21	10	1	0	0
55	2	1	0	2	12,00	3	0	0	0	0
56	2	1	0	1	233,00	50	30	1	1	1
**57**	**1**	**1**	**2**	**1**	**65,00**	**55**	**35**	**0**	**0**	**1**
58	1	1	0	1	33,00	10	5	1	1	1
59	1	1	0	3	22,00	9	6	0	0	0
60	1	1	0	2	14,00	10	0	0	0	0
**61**	**2**	**1**	**2**	**1**	**50,00**	**50**	**7**	**1**	**0**	**1**
62	2	1	0	1	53,00	41	35	1	0	1
63	2	1	0	1	35,00	20	10	1	0	0
64	2	1	0	1	31,00	15	7	1	0	0
**65**	**2**	**1**	**3**	**1**	**136,00**	**60**	**50**	**1**	**0**	**0**
66	2	1	0	1	37,00	15	0	1	0	0

67	2	1	0	1	35,00	32	20	0	1	0
68	2	1	0	1	3,00	12	0	0	0	0
**69**	**1**	**1**	**1**	**1**	**39,00**	**25**	**5**	**0**	**1**	**0**
70	1	1	0	2	48,00	21	21	0	0	0
71	1	1	0	3	26,00	12	6	0	0	1
72	1	1	0	3	37,00	8	6	0	0	1
**73**	**1**	**1**	**1**	**1**	**86,00**	**59**	**50**	**1**	**0**	**1**
74	1	1	0	2	25,00	20	0	0	0	0
75	1	1	0	3	3,00	7	2	0	0	0
76	1	1	0	3	24,00	11	6	0	0	0
**77**	**1**	**1**	**4**	**1**	**75,00**	**60**	**42**	**1**	**0**	**1**
78	1	1	0	1	14,00	12	0	0	0	0
79	1	1	0	1	42,00	7	5	1	0	0
80	1	1	0	3	1,00	8	0	0	0	0
**81**	**2**	**1**	**2**	**1**	**72,00**	**50**	**40**	**1**	**0**	**1**
82	2	1	0	3	87,00	20	20	0	0	1
83	2	1	0	1	27,00	15	5	0	0	0
84	2	1	0	3	25,00	12	0	0	0	0
**85**	**2**	**2**	**1**	**1**	**99,00**	**63**	**55**	**1**	**0**	**1**
**86**	2	2	0	1	24,00	15	5	0	0	0
87	2	2	0	1	5,00	4	1	0	0	0
88	2	2	0	1	25,00	15	5	0	0	0
**89**	**2**	**1**	**2**	**1**	**105,00**	**45**	**20**	**1**	**0**	**1**
90	2	1	0	1	25,00	20	5	0	0	0
91	2	1	0	1	32,00	20	5	0	0	0
92	2	1	0	1	8,00	10	0	0	0	0
**93**	**2**	**1**	**3**	**1**	**67,00**	**51**	**40**	**1**	**0**	**0**
94	2	1	0	3	3,00	12	0	0	0	0
95	2	1	0	1	6,00	6	0	0	0	0
96	2	1	0	1	64,00	33	30	1	0	1
**97**	**1**	**1**	**2**	**4**	**80,00**	**54**	**50**	**1**	**0**	**1**
98	1	1	0	1	87,00	40	20	1	1	1
99	1	1	0	1	2,00	11	0	0	0	0
100	1	1	0	1	28,00	20	3	1	0	0

**101**	**2**	**1**	**3**	**1**	**110,00**	**45**	**24**	**1**	**0**	**1**
102	2	1	0	1	146,00	32	25	1	0	0
103	2	1	0	1	36,00	31	0	1	0	0
104	2	1	0	1	68,00	50	30	1	0	1
**105**	**2**	**1**	**3**	**1**	**154,00**	**52**	**51**	**1**	**0**	**1**
106	2	1	0	3	22,00	10	4	0	0	0
107	2	1	0	1	1,00	5	0	0	0	0
108	2	1	0	2	29,00	4,5	4,5	0	0	0
**109**	**2**	**1**	**1**	**1**	**70,00**	**40**	**30**	**1**	**0**	**0**
110	2	1	0	1	28,00	18	18	0	0	0
111	2	1	0	1	57,00	14	7	1	0	1
112	2	1	0	1	32,00	22	11	0	0	0
**113**	**1**	**1**	**1**	**1**	**75,00**	**37**	**30**	**1**	**1**	**1**
114	1	1	0	1	28,00	8	8	1	1	0
115	1	1	0	1	64,00	17	1	1	0	1
116	1	1	0	2	9,00	1,8	0	0	0	0
**117**	**2**	**1**	**5**	**1**	**78,00**	**35**	**4**	**1**	**1**	**1**
118	2	1	0	1	19,00	15	0	0	0	0
119	2	1	0	2	9,00	53	0	0	0	0
120	2	1	0	2	19,00	2,3	1,5	0	0	1
**121**	**2**	**1**	**3**	**1**	**81,00**	**53**	**44**	**1**	**0**	**1**
122	2	1	0	1	8,00	12	0	0	0	0
123	2	1	0	1	27,00	8	0	0	0	0
124	2	1	0	3	20,00	11	11	0	0	1
**125**	**1**	**1**	**2**	**1**	**61,00**	**49**	**20**	**1**	**1**	**0**
126	1	1	0	1	67,00	62	45	1	1	0
127	1	1	0	1	37,00	12	10	1	0	0
128	1	1	0	1	30,00	15	15	0	0	0
Bold and centred :	1-COUBERT			1-THERMAL			1-PMR			0- NO
Non bold and righ	2-LA MUSSE			2-ELECTRIC			2-HOME			1-YES
MD:Missing Data				3-CHEMICAL						
				4-RADIOLOGICAL						

Associated TBI	Associated SCI	Blast	Psychol tbles	Ventilation period	Sedation period	Incision of Discharge	Dialysis	Prolonged immobilizati on (days)	Difficulties for sedation
**0**	**0**	**0**	**0**	**173**	**166**	**1**	**0**	**173**	**1**
0	0	0		6	1	0	0	0	0
0	0	0	0	0	0	0	0	0	0
0	0	1	0	0	0	0	0	0	0
**0**	**0**	**0**	**1**	**50**	**30**	**1**	**0**	**15**	**1**
0	0	0	0	0	832	0	0	0	0
0	0	0	0	0	6	0	0	0	0
0	0	0	0	0	2	0	0	0	0
**0**	**0**	**0**	**1**	**100**	**51**	**1**	**1**	**96**	**0**
0	0	0	0	1	1	0	0	0	0
0	0	0	0	90	77	1	1	52	0
0	0	0	1	57	55	1	0	0	0
**0**	**0**	**0**	**1**	**46**	**32**	**1**	**0**	**0**	**1**
1	0	0	1	37	24	0	0	0	0
0	0	0	1	0	7	0	0	6	0
0	0	0	1	32	31	0	0	0	0
**1**	**0**	**0**	**0**	**58**	**49**	**1**	**0**	**37**	**0**
0	0	0	0	4	2	0	0	0	0
0	0	0	0	0	1	0	0	0	0
0	0	0	0	0	0	0	0	0	0
**0**	**0**	**0**	**1**	**96**	**91**	**1**	**0**	**77**	**1**
0	0	0	0	0	0	0	0	0	0
0	0	0	0	38	51	0	0	26	0
0	0	0	0	0	18	1	0	0	0
**0**	**0**	**0**	**1**	**62**	**35**	**1**	**0**	**73**	**0**
0	0	0	0	0	4	0	0	MD	0
0	0	0	0	0	4	0	0	0	1
0	0	0	0	0	0	0	0	0	0
**0**	**0**	**0**	**0**	**82**	**58**	**1**	**1**	**0**	**0**
0	0	0	0	48	46	1	0	39	0
0	0	0	0	0	9	0	0	0	0
0	0	0	0	0	1	0	0	0	0

**0**	**0**	**0**	**1**	**118**	**69**	**0**	**1**	**0**	**0**
0	0	0	1	7	16	0	0	MD	0
0	0	0	0	0	0	0	0	0	0
0	0	0	0	0	8	0	0	0	0
**0**	**0**	**0**	**0**	**0**	**0**	**1**	**0**	**0**	**0**
0	0	0	0	21	26	1	0	MD	1
0	0	0	0	0	15	1	0	0	0
0	0	0	0	0	8	0	0	7	0
**1**	**0**	**1**	**1**	**111**	**56**	**0**	**1**	**101**	**0**
0	0	0	0	60	91	1	1	MD	1
0	0	0	0	1	1	1	0	0	0
0	0	0	0	0	0	0	0	0	0
**0**	**0**	**0**	**0**	**168**	**204**	**1**	**0**	**221**	**0**
0	0	0	0	0	1	0	0	0	0
0	0	0	0	0	14	0	0	0	0
0	0	0	1	0	1	0	0	0	0
**0**	**0**	**0**	**1**	**83**	**44**	**1**	**0**	**88**	**1**
0	0	0	0	0	0	0	0	0	0
0	0	0	1	0	0	0	0	0	0
0	0	0	0	0	28	0	0	0	0
**0**	**0**	**0**	**1**	**48**	**48**	**1**	**1**	**41**	**0**
0	0	0	0	51	52	1	0	0	0
0	0	0	0	0	0	0	0	0	0
0	0	0	1	79	MD	1	1	MD	0
**0**	**0**	**0**	**0**	**54**	**53**	**1**	**0**	**52**	**0**
0	0	0	1	28	19	1	0	0	0
0	0	0	1	0	16	0	0	0	0
0	0	0	0	0	0	0	0	13	0
**0**	**0**	**0**	**0**	**39**	**38**	**1**	**0**	**MD**	**0**
0	0	0	1	46	51	0	0	51	0
0	0	0	1	14	13	0	0	0	0
0	0	0	0	1475	16	0	0	0	1
**0**	**0**	**0**	**1**	**290**	**88**	**1**	**1**	**MD**	**1**
0	0	0	0	32	31	0	1	0	0

0	0	0	0	0	29	1	0	23	0
0	0	0	0	0	0	0	0	0	0
**0**	**0**	**0**	**0**	**1**	**33**	**0**	**0**	**0**	**0**
0	0	0	0	0	0	0	0	0	0
0	0	0	0	0	0	1	0	0	0
0	0	0	1	5	5	0	0	0	0
**0**	**0**	**0**	**0**	**66**	**53**	**1**	**0**	**0**	**0**
0	0	0	1	0	24	0	0	24	0
0	0	0	0	0	0	0	0	0	0
0	0	0	0	0	1	1	0	0	0
**0**	**0**	**0**	**1**	**62**	**62**	**0**	**0**	**MD**	**1**
0	0	0	1	0	0	0	0	0	0
0	0	0	1	29	29	1	0	0	1
0	0	0	0	0	0	0	0	0	0
**0**	**0**	**1**	**0**	**64**	**44**	**1**	**0**	**MD**	**0**
0	0	0	1	0	0	1	0	0	0
0	0	1	0	0	18	1	0	0	0
0	0	0	0	0	3	0	0	0	0
**1**	**0**	**0**	**0**	**64**	**28**	**1**	**0**	**MD**	**0**
0	0	0	0	0	7	0	0	0	0
0	0	0	0	0	0	0	0	0	0
0	0	0	0	0	8	1	0	0	0
**0**	**0**	**0**	**1**	**100**	**100**	**1**	**0**	**24**	**1**
0	0	0	0	0	0	0	0	0	0
0	0	0	0	0	3	0	0	31	0
0	0	0	0	0	0	0	0	0	0
**0**	**0**	**1**	**0**	**57**	**39**	**1**	**0**	**MD**	**0**
0	0	0	0	0	0	0	0	0	0
0	0	0	0	0	0	0	0	0	0
0	0	0	1	39	44	1	0	0	0
**0**	**0**	**0**	**0**	**63**	**790**	**1**	**0**	**MD**	**0**
0	0	0	0	60	60	1	0	28	0
0	0	0	0	0	0	0	0	0	0
0	0	0	0	2	23	1	0	0	0

**0**	**0**	**0**	**1**	**65**	**33**	**1**	**0**	**0**	**0**
0	0	0	1	131	69	1	0	0	0
1	0	0	0	13	16	0	0	0	0
1	0	0	0	57	62	1	0	85	1
**0**	**0**	**0**	**0**	**116**	**75**	**1**	**0**	**MD**	**0**
0	0	0	0	0	0	0	0	0	0
0	0	1	0	0	0	0	0	0	0
0	0	0	0	0	0	0	0	0	0
**0**	**0**	**0**	**1**	**65**	**36**	**0**	**0**	**49**	**0**
0	0	1	1	0	0	0	0	0	0
0	0	0	0	44	52	1	1	0	0
0	0	0	0	0	0	0	0	28	0
**0**	**0**	**0**	**1**	**74**	**20**	**0**	**1**	**49**	**0**
0	0	0	1	2	19	0	0	0	0
0	0	0	1	0	7	0	0	0	0
0	0	0	0	0	0	0	0	0	0
**0**	**0**	**0**	**0**	**68**	**35**	**0**	**1**	**48**	**0**
0	0	0	0	0	1	0	0	0	0
0	0	0	0	0	0	0	0	0	0
0	0	0	0	0	0	0	0	0	0
**0**	**0**	**0**	**1**	**74**	**41**	**1**	**1**	**MD**	**1**
0	0	1	0	0	0	0	0	0	0
0	0	0	0	0	1	0	0	0	0
0	0	0	0	0	0	0	0	7	0
**0**	**0**	**0**	**1**	**45**	**48**	**1**	**0**	**41**	**0**
0	0	0	0	46	48	1	0	34	0
0	0	0	1	7	7	0	0	MD	0
0	0	0	0	0	7	0	0	14	0
0- NO	0- NO	0- NO	0- NO	0- NO	0- NO			0- NO	0- NO
1-YES	1-YES	1-YES	1-YES	1-YES	1-YES			1-YES	1-YES

agitation	Follow up (days)	Curarization	Cumulative duration of curarization	Inhalation lesions
**1**	**942**	1	32	**1**
0	13	0	0	0
0	9	0	0	0
0	13	0	0	0
**1**	**634**	1	1	**1**
0	93	0	0	0
0	26	0	0	0
0	2	0	0	0
**0**	**334**	1	3	**1**
0	9	0	0	0
0	174	1	25	1
0	190	1	7	1
**1**	**1380**	0	0	**1**
1	133	1	1	1
0	24	0	0	0
0	163	0	0	1
**0**	**147**	0	0	**0**
0	29	0	0	0
0	49	0	0	0
0	1	0	0	0
**0**	**597**	1	1	**0**
0	10	0	0	0
0	102	0	0	0
0	317	0	0	0
**0**	**439**	0	0	**0**
0	44	0	0	0
1	363	0	0	1
0	533	0	0	0
**0**	**350**	1	2	**1**
1	158	1	4	1
0	30	0	0	0
0	7	0	0	0

**1**	**793**	1	4	**1**
0	138	0	0	0
0	40	0	0	0
0	359	0	0	MD
**0**	**243**	1	DM	**MD**
1	417	0	0	0
0	105	0	0	0
0	119	0	0	0
**0**	**281**	1	7	**1**
1	128	1	4	0
0	118	0	0	1
0	132	0	0	0
**0**	**669**	1	14	**1**
0	738	0	0	0
0	609	0	0	0
0	115	0	0	0
**0**	**179**	1	4	**1**
0	13	0	0	0
0	327	0	0	0
1	302	0	0	1
**0**	**169**	1	12	**0**
1	399	1	16	0
0	30	0	0	0
1	295	1	15	1
**0**	**928**	0	0	**0**
0	95	0	0	0
0	134	0	0	0
0	32	0	0	0
**0**	**173**	1	6	**1**
1	142	0	0	0
0	117	0	0	1
1	100	1	1	1
**0**	**601**	1	2	**1**
0	46	1	14	1

0	126	0	0	0
0	5	0	0	0
**0**	**127**	0	0	**0**
0	559	0	0	0
0	47	0	0	0
1	106	0	0	0
**0**	**416**	1	1	**1**
0	243	0	0	0
0	5	0	0	0
0	113	0	0	0
**1**	**1220**	1	6	**1**
0	18	0	0	0
1	357	0	0	0
0	7	0	0	0
**0**	**569**	1	8	**0**
1	504	0	0	0
0	391	0	0	0
0	27	0	0	0
**0**	**972**	1	5	**1**
0	47	0	0	0
0	12	0	0	0
0	169	0	0	0
**0**	**481**	0	0	**1**
0	112	0	0	0
0	121	0	0	0
0	9	0	0	0
**0**	**210**	1	1	**1**
0	5	0	0	0
0	6	0	0	0
0	152	0	0	1
**0**	**614**	1	9	**0**
1	171	1	1	1
0	10	0	0	0
0	207	1	1	0

**0**	**242**	1	3	**1**		
1	1134	1	1	1		
0	113	0	0	1		
0	68	0	0	1		
**0**	**496**	1	7	**1**		
0	88	0	0	0		
0	7	0	0	0		
0	125	0	0	0		
**0**	**147**	1	10	**1**		
0	114	0	0	0		
1	131	1	6	0		
0	128	0	0	0		
**1**	**184**	1	1	**1**		
0	99	0	0	0		
0	307	0	0	0		
0	79	0	0	0		
**0**	**170**	1	7	**0**		
0	22	0	0	0		
0	13	0	0	0		
0	294	0	0	0		
**0**	**165**	1	2	**1**		
0	23	0	0	0		
0	113	0	0	0		
0	38	0	0	0		
**0**	**156**	0	0	**0**		
1	158	1	3	1		
1	125	0	0	1		
0	216	0	0	0		
	0- NO	0- NO		0- NO		0- NO
	1-YES	1-YES		1-YES		1-YES
