# Supplementary material for: Two new risk factors for heterotopic ossification development after severe burns
Source: PLoS One. 2017 Aug 4;12(8):e0182303. doi: 10.1371/journal.pone.0182303 (PMC5544177; doi:10.1371/journal.pone.0182303)
Supplement: S2 Table — (PDF) [file pone.0182303.s002.pdf]

HO CASE	délay from accident to diagnosis/j	Localization	Side	Main localization around the joint	Localization under a burn zone	Discharge incision in regard of the HO
1	117	1	1	2	1	1
2	159	1	2	2	1	1
3	159	4	1	4	1	1
4	159	4	2	4	1	1
5	410	3	1	7	1	0
6	410	2	1	MD	1	0
7	410	2	2	MD	1	0
8	114	1	1	2	1	1
9	58	1	1	1	1	0
10	53	1	1	MD	1	0
11	147	1	2	MD	1	0
12	147	3	1	MD	1	0
13	118	1	1	7	1	1
14	70	1	1	10	1	1
15	70	1	2	2	1	1
16	70	4	2	1	1	1
17	77	3	1	MD	0	0
18	70	2	2	MD	0	0
19	95	1	1	2	1	1
20	95	1	2	2	1	1
21	95	3	1	7	0	0
22	95	3	2	7	0	0
23	91	1	2	2	1	1
24	170	2	1	3	1	0
25	170	2	2	3	1	0
26	46	1	1	7	1	1
27	121	3	1	2	1	0
28	242	1	1	2	1	1
29	242	1	2	2	1	1
30	107	4	1	4	1	0
31	107	4	2	10	1	1
32	121	1	1	2	1	0
33	118	2	2	9	1	1
34	58	1	1	2	0	0
35	58	1	2	2	0	0
36	54	1	1	6	1	1
37	54	1	2	6	1	1
38	136	1	1	6	1	1
39	136	1	2	6	1	1
40	138	4	1	5	1	1
41	39	1	2	MD	1	0
42	91	1	1	6	1	1
43	69	1	1	6	1	0

44	69	1	2	MD	1	0
45	69	4	1	MD	1	0
46	69	4	2	1	1	0
47	72	1	1	6	1	1
48	72	1	2	6	1	1
49	92	1	2	8	1	1
50	45	1	1	7	1	1
51	45	5	1	7	1	1
52	69	1	1	8	1	0
53	69	2	1	4	1	1
54	69	2	2	4	1	1
55	88	1	1	2	1	0
56	88	1	2	2	1	0
57	116	1	2	6	1	0
58	116	4	1	4	1	0
59	116	2	2	MD	1	0
60	71	4	2	4	1	0
61	76	2	1	1	0	0
62	79	1	1	7	1	0
63	79	1	2	2	1	0
64	79	4	2	5	1	0
65	92	2	2	3	0	0
66	92	3	1	7	0	0
67	82	4	1	4	1	1
68	82	4	2	4	1	1
69	82	3	1	7	1	0
70	62	1	1	4	1	1
71	62	4	1	4	1	1
72	166	4	2	4	1	1
73	166	2	1	MD	0	0
74	166	2	2	8	0	0
		1-Elbow	1-Left	1-Anterior	0-No	0-No
		2-Hip	2-Right	2-Posterior	1-Yes	1-Yes
		3-Knee		3-Superior		
		4-Shoulder		4-Inferior		
		5-Wrist		5-Posterior & inferior		
				6-Posterior & medial		
				7-Medial		
				8-Posterior & lateral		
				9-Lateral		
				10-Other		

Condition for diagnosis	Associated clinical symptoms	ROM before treatment	use of NSAID	Use of Bisphophonates	Mobilization by a trained physiotherapist (gentle)
0	1	70/-70/-20/25	0	0	1
0	1	70/-70/50/15	0	0	1
0	1	30/10/50/-15/-45/65	0	0	1
0	1	25/20/45/-10/-45/60	0	0	1
0	3	30/0	0	0	1
0	3	70/-10/20/20/15/35	0	0	1
0	3	75/-10/30/25/30/30	0	0	1
1	1	MD	0	0	1
1	0	90/-45/MD/MD	0	0	1
2	0	MD	0	0	1
1	0	45/-10/MD/MD	0	0	1
1	0	65/0/0	0	0	1
1	2	130/-40/MD/MD	0	0	1
1	0	55/-40/30/30	0	0	1
1	2	55/-40/20/60	0	0	1
1	0	15/5/30/MD/5	0	0	1
1	0	50/-20/0	0	0	1
1	0	90/MD/20/10/MD/MD	0	0	1
1	1	90/10/MD/MD	0	0	1
1	1	90/10/MD/MD	0	0	1
1	1	40/0/0	0	0	1
1	1	40/0/0	0	0	1
2	3	90/-40/MD/MD	0	0	1
0	3	0/60/5/20/10/10	0	0	1
0	3	0/60/5/25/0/20	0	0	1
1	0	90/-20/MD/MD	0	0	1
1	0	80/0/0	0	0	1
1	0	70/-10/MD/MD	0	0	1
1	0	45/-25/MD/MD	0	0	1
1	0	20/30/45/-15/-50/70	0	0	1
1	0	30/45/60/15/-40/75	0	0	1
1	0	145/-5/60/60	0	0	1
2	0	5/115/35/25/30/20	0	0	1
1	0	130/-60/90/90	0	0	1
1	0	45/-45/90/90	0	0	1
1	0	50/-40/70/45	0	0	1
1	0	60/-40/70/60	0	0	1
1	2	90/60/MD/MD	0	0	1
1	0	90/-60/MD/MD	0	0	1
1	0	60/MD/60/D/MD/MD	0	0	1
1	1	80/-20/MD/MD	0	0	1
1	2	90/- 50/90/90	0	0	1
1	1	MD	0	0	1

1	1	MD	0	0	1
1	1	MD	0	0	1
1	1	MD	0	0	1
1	2	90/-20/70/20	0	0	1
1	2	100/-10/70/10	0	0	1
1	2	50 /-40/90/90	0	0	1
1	2	25/-25/MD/MD	0	0	1
0	3	20/30/75/-60/15/15	0	0	1
0	3	70/-35/65/50	0	0	1
0	0	30/85/30/30/35/30	0	0	1
0	0	15/105/35/35/40/30	0	0	1
1	0	140/-30/90/70	0	0	1
1	0	100/-60/90/70	0	0	1
1	2	60/70/90/10	0	0	1
1	0	MD/MD/80/MD/MD/MD	0	0	1
0	0	0/50/MD/MD/MD/MD	0	0	1
0	3	70/30/70/10/15/70	0	0	1
0	0	15/100/30/30/25/30	0	0	1
0	0	85/-25/65/50	0	0	1
0	0	85/-15/90/50	0	0	1
0	0	80/25/80/0/5/55	0	0	1
0	0	25/105/20/50/40	0	0	1
0	0	80/-15/0	0	0	1
0	0	35/35/60/0/0/70	0	0	1
0	0	MD	0	0	1
0	0	75/-10/0	0	0	1
0	0	90/-15/50/60	0	0	1
0	0	70/25/10/70/60/15	0	0	1
1	0	MD/MD/60/MD/MD/MD	0	0	1
1	0	MD/90/MD/MD/MD/MD	0	0	1
1	1	MD/70/MD/MD/MD/MD	0	0	1
0- Systematical	1-Pain		0-No	0-No	0-No
1-Loss of ROM	2-Nerve compression		1-Yes	1-Yes	1-Yes
2-Pain	Loss of range of motion				

Surgery for removal	Surgical indication	ROM after treatment	Follow up (Days)
1	1	110/-10/0/0	942
1	1	MD	942
0	MD	110/75/105/15/-10/80	942
0	MD	80/25/75/-5/-20/80	942
0	MD	95/0/0	942
0	MD	MD/90/MD/MD/MD/MD	942
0	MD	MD/90/MD/MD/MD/MD	942
1	1	MD	334
0	MD	90/-60/55/70	634
0	1	MD	
MD	MD	MD	147
MD	MD	MD	147
1	1	130/-25/MD/MD	597
0	1	110/-45/90/80	439
1	2+3	90/-45/90/80	439
0	1	35/MD/40/MD/0/15	439
0	1	90/0/0	439
0	1	15/130/45/20/45/35	439
0	1	100/-20/MD/MD	350
0	1	100/-20/MD/MD	350
0	1	100/0/0	350
0	1	130/0/0	350
1	1	100/-50/MD/MD	793
0	1	0/95/0/25/5/25	793
0	1	0/100/10/25/5/15	793
0	1	90°/0/MD/MD	243
0	1	130/0/0	274
0	1	150/0/MD/MD	712
0	1	150/O/MD/MD	669
0	MD	20/25/50/0/0/80	288
0	MD	60/40/60/15/5/90	288
0	1	145/-5/60/60	169
0	3	10/105/30/20/35/20	169
1	1	135/-5/90/90	928
1	1	130/-10/90/90	928
1	1	110/-25/90/80	173
0	1	130/-25/90/80	173
0	4	100/-30/MD/MD	601
0	4	120/-30/MD/MD	601
0	1	90/MD/90/MD/MD/MD	601
0	MD	90/-60/MD/MD	127
1	1	130/-10/90/80	416
1	1	90/-10/90/80	1220

1	1	130/-30/MD/MD	1220
1	1	MD	1220
1	1	MD	1220
1	2	120/-M/MD10/	569
1	2	120/-20/MD/MD	569
1	2	100/-20/90/80	972
1	1	115/-30/MD/MD	481
0	1	90/10/15/20	481
0	MD	110/-60/90/80	210
0	MD	15/115/40/35/45/35	210
0	MD	5/120/45/35/50/35	210
0	1	140/-30/90/70	614
1	1	115/-20/MD/MD	614
1	1	MD	
0	1	MD	
0	1	MD	
0	1	120/60/110/35/80/MD	150
0	1	15/120/45/35/35/35	184
0	MD	90/-25/65/60	174
0	MD	85/-15/65/60	174
0	MD	90/25/80/0/5/55	174
0	MD	0/105/25/20/50/40	174
0	MD	80/-5/0	174
0	1	MD	
0	1	MD	
0	1	MD	
0	1	100/-20/55/45	156
0	1	60/40/50/0/30/75	156
0	1	130/MD/130/MD/MD/MD	496
0	1	-20/100/45/30/20/20	496
0	1	15/130/45/15/45/35	496
0-No	1-Loss of ROM		
1-Yes	2-Nerve compression		
3- Spontaneous resolution			
4- Oppisition from the patient			
